# Parasites of an Arctic scavenger; the wolverine (*Gulogulo*)

**DOI:** 10.1016/j.ijppaw.2020.10.004

**Published:** 2020-10-16

**Authors:** Sophie E. Watson, Frank Hailer, Nicolas Lecomte, Pratap Kafle, Rajnish Sharma, Emily J. Jenkins, Malik Awan, Vincent L’Hérault, Sarah E. Perkins

**Affiliations:** a School of Biosciences, The Sir Martin Evans Building, Museum Avenue, Cardiff University, Cardiff, Wales, UK; b Université de Moncton, Campus de Moncton, Pavillon Léopold-Taillon, 18, Avenue Antonine-Maillet, Moncton, New Brunswick, Canada; c Department of Veterinary Microbiology, Western College of Veterinary Medicine, University of Saskatchewan, Saskatoon, Saskatchewan, Canada; d Department of Environment, Government of Nunavut, PO Box 209, Igloolik, Nunavut, Canada; e ArctiConnexion, Québec, Canada

**Keywords:** Wolverine, Helminths, Arctic, Parasite community, 18S sequencing, Parasite surveillance

## Abstract

Parasites are fundamental components within all ecosystems, shaping interaction webs, host population dynamics and behaviour. Despite this, baseline data is lacking to understand the parasite ecology of many Arctic species, including the wolverine (*Gulogulo*), a top Arctic predator and scavenger. Here, we combined traditional count methods (i.e. adult helminth recovery, where taxonomy was confirmed by molecular identification) with 18S rRNA high-throughput sequencing to document the wolverine parasite community. Further, we investigated whether the abundance of parasites detected using traditional methods were associated with host metadata, latitude, and longitude (ranging from the northern limit of the boreal forest to the low Arctic and Arctic tundra in Nunavut, Canada). Adult parasites in intestinal contents were identified as *Baylisascaris devosi* in 72% (n = 39) of wolverines and *Taenia* spp. in 22% (n = 12), of which specimens from 2 wolverines were identified as *T. twitchelli* based on COX1 sequence. 18S rRNA high-throughput sequencing on DNA extracted from faeces detected additional parasites, including a pseudophyllid cestode (*Diplogonoporus* spp. or *Diphyllobothrium* spp.), two metastrongyloid lungworms (*Angiostrongylus* spp. or *Aelurostrongylus* spp., and *Crenosoma* spp.), an ascarid nematode (*Ascaris* spp. or *Toxocara* spp.), a *Trichinella* spp. nematode, and the protozoan *Sarcocystis* spp., though each at a prevalence less than 13% (n = 7). The abundance of *B. devosi* significantly decreased with latitude (slope = -0.68; R^2^ = 0.17; P = 0.004), suggesting a northerly limit in distribution. We describe *B. devosi* and *T. twitchelli* in Canadian wolverines for the first time since 1978, and extend the recorded geographic distribution of these parasites ca 2000 km to the East and into the tundra ecosystem. Our findings illustrate the value of molecular methods in support of traditional methods, encouraging additional work to improve the advancement of molecular screening for parasites.

## Highlights

• Combining traditional and molecular methods better captures parasite diversity.
• *B. devosi* and *Taenia* spp. distribution extends ca 2000 km East and into the tundra.
• The abundance of *B. devosi* in wolverines significantly decreases with latitude.
• *B. devosi* and *Taenia* spp. abundance is not associated with wolverine host metadata.

## Graphical abstract

**Figure:**
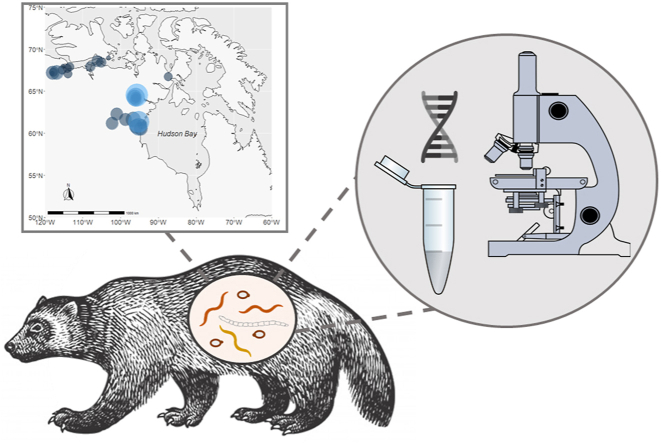

## 1 Introduction

Representing over 50% of all organisms on Earth, parasites are a fundamental component within all ecosystems –shaping interaction webs as well as host population dynamics and behaviour (Dobson et al., 2008; Hudson et al., 2006; Marcogliese, 2001). Even at high latitudes where biological diversity is purported to be generally low (such as the Arctic), parasite communities can be diverse and often more species-rich than those of their vertebrate hosts (Hoberg et al., 2008; Kutz et al., 2009). For example, over 60 species of parasite are described in four ungulate species of High Arctic Greenland and North America (Kutz et al., 2012). Such descriptions highlight the extensive distribution of parasites throughout Arctic hosts.

The Arctic is undergoing some of the most rapid rates of climate change and is therefore at high risk of parasite emergence, which may shift baseline data (Brooks and Hoberg, 2007; Kutz et al., 2009; McLaughlin, 2011). It has been forecast that increases in air temperature will be most dramatic at high latitudes (Dobson et al., 2015) and already the Arctic has experienced the 10 warmest years in the past 2 millennia (Kaufman et al., 2009; Hoegh-Guldberg et al., 2018). One expected consequence of warming air temperatures and increased precipitation in the northern hemisphere is a significant increase in the northern expansion of parasites and their hosts into regions that were previously inhospitable to them (Brooks and Hoberg, 2007; Kutz et al., 2009; McLaughlin, 2011). Consequently, the monitoring of wildlife plays an important role in identifying changes such that actions can be taken to mitigate or minimize pressure. Despite this, there remains a lack of baseline data regarding the parasite ecology for many Arctic species, including the wolverine (*Gulo* *gulo*), a top Arctic predator and scavenger.

Helminths have previously been recovered from wolverines of Alaska and the Northwest Territories of Canada, including; *Alaria* sp., *Taenia martis*, *T. twitchelli*, *Diphyllobothrium* sp., *Physaloptera* sp., *Baylisascaris devosi*, *Mesocestoides kirbyi* and *Molineus patens* (Addison and Boles, 1978; Rausch, 1954), though, to the best of our knowledge, these parasites have not been reported or surveyed in wolverines in over 40 years. Sequences for *B. devosi* from Canadian wolverines are, however, present on Genbank, as uploaded by Gesy et al., in 2015 (Accession number: KM216978 to 985). Additionally, *Trichinella* infection has been widely reported in wolverines across their entire range (Reichard et al., 2008b; Sharma et al, 2019c, 2020) with the highest prevalence (88%) being reported in 41 wolverines from Nunavut (Reichard et al., 2008b), and two species of the Apicomplexan, *Sarcocystis* have been reported in wolverines from Nunavut, Canada (Dubey et al., 2010); sarcocysts were recovered from 33 of 41 (80%) wolverines screened (Dubey et al., 2010). Additionally, *Toxoplasma gondii* infection has been documented in wolverines from the Northwest Territories, the Yukon, Nunavut and British Columbia (Philippa et al., 2004; Reichard et al., 2008a; Sharma et al., 2019a, 2019b). Not only is parasite surveillance in wolverines limited, but there appears to be a paucity of information related to how parasitic infections of wolverines may be associated with host and geographic metadata.

The wolverine (family: Mustelidae) occupies a heterogeneous geographic range that covers northern Arctic tundra, taiga, mountain, and boreal forest ecosystems (Copeland et al., 2010; Dawson et al., 2010). Typically, due to ecological conditions, parasite species richness in carnivores typically decreases on a latitudinal gradient from south to north (Lindenfors et al., 2007), though there is a lack of comprehensive baselines for parasite diversity in Arctic hosts (Meltofte et al., 2013). It might therefore be expected that wolverines occupying a more northerly region of their geographic range may host a diversity of parasites that is less species rich compared to more southerly inhabitants. Across the varied landscapes they occupy, wolverines can travel huge distances each year (Copeland et al., 2010; Dawson et al., 2010), with male wolverines (which are the larger sex (Banci, 1994)) occupying up to ~2500 km^2^, compared to the smaller range of females at ~400 km^2^ (Dawson et al., 2010). Across multiple mammalian taxa, where sexual size dimorphism is male-biased, so too is parasitism (Moore and Wilson, 2002; reviewed in Poulin and Morand 2004). Similarly, male bias in home range size can also lead to disparity in infection between sexes (reviewed in Poulin and Morand 2004). A large home range equates to an increased overlap with other host species and environmental conditions, which can expose a host to a broad diversity of parasites (reviewed in Poulin and Morand 2004; Leung and Koprivnikar 2016; Becker et al., 2018). As such, prevalence and intensity of parasitic infection is commonly, but not exclusively, higher in male mammalian hosts compared to females (Poulin, 1996; Zuk and McKean, 1996). Similarly, prevalence and intensity of parasitic infection typically differs between adult compared to juvenile hosts, though this varies depending on host and the conditions of infection (Woolhouse, 1998). Across their large home range, wolverines consume a broad range of prey items. As facultative scavengers, wolverines consume a range of species, including moose (*Alces alces*), caribou (*Rangifer tarandus*), muskoxen (*Ovibos moschatus*), hare (*Lepus* sp.) Arctic ground squirrels (*Spermophilus parryii*), voles and lemmings (Muridae), ptarmigan (*Lagopus* sp.), seal (*Phoca* sp.) and migratory bird species (Koskela et al., 2013; L’Hérault et al., 2018; Lofroth et al., 2007). Hosts with a broad diet are potentially exposed to a larger variety of trophically transmitted parasites (Anderson and Sukhdeo, 2011; Aponte et al., 2014; Vitone et al., 2004). A generalist diet in wolverines may therefore equate to high parasite exposure.

Here we use both a traditional parasite count method (i.e. adult helminth recovery based on gross examination of intestinal contents, where taxonomy was confirmed by molecular identification) and 18S rRNA high-throughput sequencing to characterise the parasite community of wolverines from Nunavut, Canada. Secondly, we investigate whether the abundance of the most common parasites; *Baylisascaris devosi* and *Taenia* spp. were associated with the following metadata: wolverine sex, age class, body length, carcass mass, latitude, and longitude (ranging from the tree line to low Arctic and Arctic tundra). We additionally determine whether a coinfection with both *B. devosi* and *Taenia* spp. (i.e. 0, 1, or ≥2 parasite species present) is associated with our wolverine metadata.

## 2 Materials and methods

### 2.1 Wolverine sampling

**Fig. 1:**
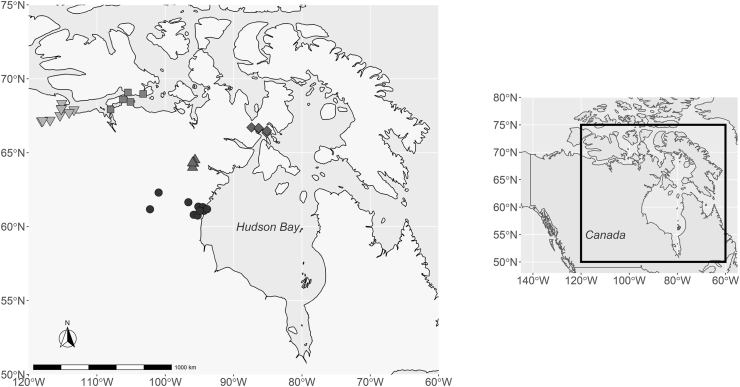
Map of individual wolverine hunting locations close by Inuit communities (circles = Arviat, triangles = Baker Lake, diamonds = Repulse Bay (Naujaat), squares = Cambridge Bay, inverse triangles = Kugluktuk) in Nunavut, Canada, 2010–2013. Inset shows sampling location (black outline) on a map of North America.

As part of a wolverine carcass collection programme initiated by the Government of Nunavut Department of Environment, 54 skinned wolverine carcasses (legally harvested for purposes other than research) were obtained from local Inuit hunters with the assistance of Hunters and Trappers Organizations (HTOs) of Nunavut, Canada. Wolverines were harvested between November 1st and April 30th from 2010 through 2013 at five distinct geographical locations representing different Inuit communities; Arviat (61 °10′N/94 °06′W; n = 17), Baker Lake (64 °31′N/96 °02′W; n = 9), Repulse Bay (Naujaat) (66 °52′N/82 °24′W; n = 7), Kugluktuk (67 °82′N/115 °09′W; n = 11), and Cambridge Bay (69 °11′N/105 °05′W; n = 10) (Fig. 1). The skinned wolverines were delivered to community conservation offices where carcasses were frozen at −20 °C and the following information was recorded: kill date, location, sex (male, 37; female, 17) and age (yearlings, 18; juveniles, 16; adults, 20). To determine the age, a lower canine from each individual was submitted to Matson's Laboratory LLC (Milltown, MT, USA) for analysis. Following Banci and Harestad (1988) and Vangen et al. (2001) individuals were then grouped into three age classes: juvenile (0–1 year, date of birth is set to March 1st), yearling (1–2 years) and adult (≥2 years). Necropsies were performed to collect the gastrointestinal tracts used within this study. All gastrointestinal tracts were shipped to Université de Moncton, Canada, January 2018.

### 2.2 Processing/dissection of gastrointestinal tracts

Dissections were conducted on gastrointestinal tracts of 54 wolverines (pylorus to anus, excluding the stomach) at the Université de Moncton (February 2018). Each intestinal tract was partially defrosted until pliable. A faecal sample and a small intestine sample were collected from near the rectum of each individual (54 faecal and 54 small intestine samples; 108 samples total) and immediately frozen at −20 °C for later 18S parasite profiling. Starting from the stomach end, the intestinal wall of the tract was then systematically cut open, washed through a series of sieves (minimum mesh size of 0.01 mm), and examined for intestinal helminths by naked eye and then using a 40X hand lens. The mucosa was scraped with a spatula into a Petri dish and also examined. Any helminths discovered were removed, counted, and stored in 70% ethanol at −20 °C until further analysis.

### 2.3 Identification of adult parasites using COX1

In total, 44 recovered adult helminths from 34 hosts were sent for identification at the Jenkins Lab, Department of Veterinary Microbiology, Western College of Veterinary Medicine, University of Saskatchewan, via molecular methods. DNA from a subset of representative specimens was extracted individually using the DNeasy Blood and Tissue Kit (Qiagen, Toronto, Canada) following manufacturer instructions. The primer set COX-1F (5′— TTTTTTGGGCATCCTGAGGTTTAT—3′) and COX-1R (5′— TAAAGAAAGAACATAATGAAAATGY —3’) (Bowles et al., 1992) was used to target a ~366 base pair region of the cytochrome *c* oxidase subunit 1 (COX1) mitochondrial gene following methods detailed in Lavikainen et al. (2003). For each sample, 25 μL reaction mix was prepared by mixing 12.5 μL 2 × Taq FroggaMix (FroggaBio, Toronto, Canada), 1 μL forward and reverse primer mixture (10 μM of each primer), 3 μL template DNA and 7.5 μL nuclease-free water. The thermal conditions used were: preheating at 94 °C for 3 min followed by 30 cycles of denaturation at 94 °C (30 s), annealing at 55 °C (30 s) and extension at 72 °C (1 min). This was followed by a 10 min final extension at 72 °C. PCR products were separated by gel electrophoresis on 1% agarose gels in TAE buffer. PCR products of 15 samples, representing samples which yeilded bands at different positions, were purified and sent for sequencing at Macrogen, South Korea. All nucleotide sequences were compared to sequences from the NCBI GenBank database using BLAST.

### 2.4 18S parasite profiling using high-throughput sequencing

Total genomic DNA was extracted from 54 faecal and small intestine samples using a QIAamp DNA Stool Mini Kit (Qiagen, Toronto, Canada). Five of the samples were extracted and processed a second time, to check for metabarcoding consistency. Methods followed the manufacturer's instructions for pathogen detection using 250 mg of sample, with the addition of a 6 min homogenization step to enhance bacterial cell lysis after the addition of buffer ASL (Step 2 in manufacturer handbook). Homogenization was achieved using a TissueLyser II (Qiagen) for 6 min at 5.5 Hz with the following combination of glass beads per tube: 0.3 g of 106 μm beads, 0.5 g of 425–600 μm beads, and x1 3 mm bead (Sigma-Aldrich, Ontario, Canada).

For parasite detection within faeces and small intestine samples, a ~260 base pair region of the V9 fragment of the 18S rRNA gene was amplified using the primer set Euk_1391f (5′— CTCAAAGATTAAGCCATGC —3′) and EukBr (5′— TTTACGGTCAGAACTAGGG —3’) (Amaral-Zettler et al., 2009) in conjunction with the mammal blocking primer: GCCCGTCGCTACTACCGATTGGIIIIITTAGTGAGGCCCT-(C3 Spacer) (Vestheim and Jarman, 2008) following methods described in the Earth Microbiome Project (http://www.earthmicrobiome.org/). In brief, targeted PCR reactions were used whereby a sequence tag was added to the 5′ end of each primer. The tag sequence was used to bind primers in a second PCR reaction during which individual sample barcodes and Illumina adapters were annealed. All barcoded products were run on 2% agarose gels. DNA concentrations of all samples were measured using PicoGreen, allowing pooling of samples at equimolar amounts. The pool (library) was cleaned using AMPure® beads. The library was then quantified using the Quant-iT™ PicoGreen® dsDNA Assay Kit (Life Technologies) and the Kapa Illumina GA with Revised Primers-SYBR Fast Universal kit (Kapa Biosystems). Average fragment size was determined using a LabChip GX (PerkinElmer) instrument. Illumina MiSeq PE250 high-throughput sequencing of 18S libraries was conducted with the MiSeq Reagent Kit v2 (500 cycles) at Génome Québec, Montréal, Canada. The raw sequencing data can be found at the National Centre for Biotechnology Information (NCBI) Sequence Read Archive (SRA) [Accession number: PRJNA662086].

### 2.5 Bioinformatic analysis

All bioinformatic analyses were conducted using QIIME2, version 2018.11 (Bolyen et al., 2018). Briefly, paired-end reads were joined using VSEARCH (Rognes et al., 2016) and quality filtered using the default settings of q-score-joined (Bokulich et al., 2013). Data reads were denoised using DADA2 (Callahan et al., 2016) with a minimum phred quality score of 28 (below which data quality tailed off). Taxonomic assignments of representative sequences from each Amplicon Sequence Variant (ASV) were performed using the SILVA (release132) reference database (Quast et al., 2013) at 99% identity so as to minimize potential identification mismatch. Taxonomic assignment was conducted in conjunction with the alignment-based taxonomy consensus classifier, BLAST+ (Camacho et al., 2009). To additionally evaluate taxonomic assignment, the Basic Local Alignment Search Tool (BLAST) (Altschul et al., 1990) was used to compare representative sequences of parasite taxa against the NCBI database. As lower taxonomic identifications are less certain, parasite identifications are reported at genus level.

### 2.6 Statistical analysis

All statistical analyses were conducted in R version 3.5.0 (R Core Team, 2018). Generalized Linear Models (GLMs) were used to investigate whether i) total *B. devosi* abundance, ii) total parasite abundance of both *B. devosi* and *Taenia* spp. and iii) coinfection of *B. devosi* and *Taenia* spp. (i.e. 0, 1, ≥2 parasite species present) were associated with sex, age class, body length, carcass mass, latitude, and longitude. An outlier, identified via a Cook's distance residuals versus leverage plot, was omitted from the models. The coinfection model did not include parasite species detected using molecular methods owing to the low prevalence identified and difficulty in distinguishing true wolverine infections from secondary parasite detection from infected individuals. To follow GLM assumption, a negative binomial error family with a log link function was used for *B. devosi* abundance and for total parasite abundance of both *B. devosi* and *Taenia* spp. A Poisson family and identity link function was used for coinfection of *B. devosi* and *Taenia* spp., as based on residual plots. No interacting terms were included due to small sample size limiting the numbers of individuals represented in each group. For 18S data, a parasitic infection was considered present if a parasite was detected in either the faecal or small intestine sample. Due to the low prevalence of detection and because the range of dietary items consumed by wolverines makes it difficult to discern whether parasite detection indicated a true infection of wolverines or is instead a secondary detection from an infected prey item, parasites detected through 18S rRNA high-throughput sequencing were not included in the statistical models.

## 3 Results

### 3.1 Parasite diversity and abundance

Based on adult helminth recovery from intestines, 83% of wolverines (n = 45) were parasitised; nine individuals had no visible parasite infection, 34 were infected with one parasite species, and 11 were coinfected. *Baylisascaris devosi* (Nematoda) was found in 72% (n = 39) of individuals at an average of 3.6 worms per individual (SD: ± 4.6, abundance range: 0–21; Fig. 2a), and *Taenia* spp. (Cestoda, O. Cyclophyllidea) were found in 22% (n = 12) individuals at an average of 1.8 worms per individual (SD: ± 6.5, abundance range: 0–45). Based on COX 1 primers, two taeniid cestodes from two hosts were identified as *T. twitchelli* (99% similarity to Genbank accession number EU544598) and seven nematodes from seven hosts were identified as *B. devosi* (99% similarity to Genbank accession number KM216978). When using 18S rRNA high-throughput sequencing, six parasite genera were detected, at a low overall prevalence (22% total, n = 12; ≤13% prevalence for any parasite). The prevalence of each parasite genus against associated host metadata can be found in Supplementary Table 1. Although a higher parasite diversity was detected using molecular methods, it is uncertain whether or not the parasites are secondary detections from infected prey items as opposed to true infections of wolverines and, in some instances, there was no consensus in taxonomic identification between the two reference databases used, and likely identifications for wolverine parasites are seldom present in these databases. For example, a metastrongyloid lungworm (detected at a prevalence of 2%; n = 1) was identified as *Angiostrongylus* sp. in accordance with the SILVA reference database (99% similarity) but *Aelurostrongylus* sp. when using the NCBI database (99.4% similarity). Similarly, an ascarid nematode was detected in 6 small intestinal and 1 faecal sample at a prevalence of 13% (n = 7), and identified as an *Ascaris* sp. in the SILVA database (99% similarity) but as *Toxocara* sp. in the NCBI database (99.4% similarity). A pseudophyllid cestode (Subclass: Eucestoda) which was identified as *Diplogonoporus* sp. according to the SILVA database (99%) or *Diphyllobothrium* sp. according to NCBI (99.4%) was detected at a prevalence of 2% (n = 1). Further, the following parasitic genera were each detected in 2% (n = 1), the identification of which were consistent between the SILVA and the NCBI databases (≥99% similarity); *Crenosoma* spp., *Trichinella* spp. (Nematoda) and *Sarcocystis* spp. (Protozoa). In addition to these 6 parasites for which wolverine could serve as potential definitive hosts, two parasites were detected which are likely parasites detected from infected prey items. For example, *Gregarina* sp. (detected in both the SILVA and NCBI databases; 99% and 96.5% respectively), a parasite of insects, was recovered at a prevalence of 7% (n = 4). Similarly, *Bodonidae* sp. (detected in both the SILVA and NCBI databases; ≥99%), an ectoparasite of fish, was recovered at a prevalence of 4% (n = 2). The *Baylisascaris* sp. and *Taenia* sp. detected using traditional methods were not detected by 18S rRNA high throughput sequencing. When using 18S rRNA sequencing, a greater diversity of parasites was recovered from faecal samples (n = 8) as opposed to small intestine samples (n = 2). However, a greater number of *Ascaris* sp. detections were recorded in small intestine samples (n = 6) compared to faecal samples (n = 1). Only *Ascaris* sp. and *Bodonidae* sp. were detected in both a faecal and a small intestine sample.

**Fig. 2a:**
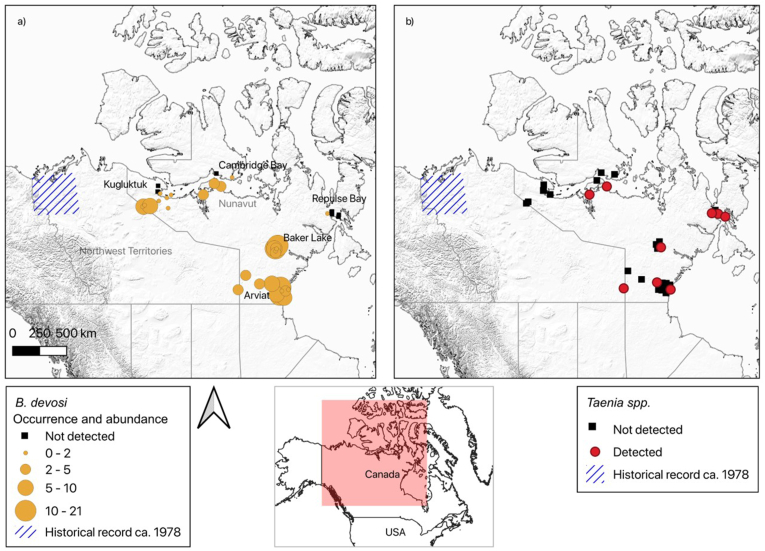
Records of *Baylisascaris devosi* in Nunavut, Canada detected within this paper (yellow), where each point is weighted by parasite abundance. Black squares represent wolverines in which *B. devosi* was not detected. Blue hashed line area indicates the previously known records of *B. devosi* reported by Addison and Bole (1978)**b)** Records of *Taenia* spp. in Nunavut, Canada detected within this paper (red). Previously known records of *Taenia* spp. (*T. twitchelli* and *T. martis*) reported by Addison and Bole (1978) are indicated by the blue hashed line area. Black squares represent wolverines in which *Taenia* spp. was not detected. (For interpretation of the references to colour in this figure legend, the reader is referred to the Web version of this article.)

### 3.2 Parasitism and host metadata

No association was found between *B. devosi* abundance and wolverine sex, age class, body length, carcass mass or longitude, but a significant decrease in *B. devosi* abundance was associated with an increase in latitude (slope = -0.68; R^2^ = 0.17; P = 0.004). Specifically, a total of 52 *B. devosi* nematodes (mean; 5.8) were recovered at 61 °N, compared to a total of 5 (mean; 1.6) at 69 °N, a mean decrease of 0.5 per degree North. The geographic region of detection for *B. devosi* recorded here extends the known distribution recorded from previous research and demonstrates the presence of this species within the Arctic tundra (Fig. 2a). Due to the number of individuals infected with *Taenia* spp. (22%, n = 12), it was not possible to compare abundance of this species alone with any host metadata; therefore both parasites were examined together (i.e. total parasite abundance). However, *T. twitchelli* is present in wolverines in a region where it was previously unrecorded, and also demonstrates the presence of this species within the Arctic tundra (Fig. 2b).

No association was found between total parasite abundance (i.e. abundance of both *B. devosi* and *Taenia* spp.) and wolverine sex, age class, body length, carcass mass, longitude or latitude (P > 0.05). Similarly, no association was found between the presence of a coinfection (0, 1 or ≥2 parasite species present) and sex, age class, body length, carcass mass, longitude or latitude (P > 0.05).

## 4 Discussion

Within this study, we combined adult helminth recovery (where taxonomy was confirmed by molecular identification using the COX1 mtDNA locus) and 18S rRNA high-throughput sequencing of DNA extracted from faecal and small intestinal samples to provide comprehensive insight into the gastrointestinal parasite community of wolverines. Two parasite species were detected by adult helminth recovery followed by molecular identification, *Baylisascaris devosi* and *Taenia twitchelli*, both of which have been recovered in wolverines previously, although the latest published study to do so is over 40 years old (Addison and Boles, 1978). Sequences for *B. devosi* from morphologically confirmed specimens from Canadian wolverines are, however, present in Genbank (Accession numbers KM216978-985). In addition, 18S rRNA targeted high-throughput sequencing detected threefold the diversity of parasite species (n = 6) compared to gross adult parasite recovery (n = 2), suggesting that combined molecular and adult parasite recovery represents an important tool for characterising the parasite community of a given host. Comparative studies in other species, including wild rats (*Rattus norvegicus* and *R. rattus*), found 18S rRNA targeted high-throughput sequencing to be at least as sensitive as traditional count methods (Hino et al., 2016; Tanaka et al., 2014), and detects a broad diversity of intestinal eukaryotes in long-tailed macaques (*Macaca fascicularis*) and spotted hyenas (*Crocuta crocuta*) (Heitlinger et al., 2017; Wilcox and Hollocher, 2018).

Although the methods used within this study offer an insight into the diversity of parasites in wolverines, it is important to note that the results may not reflect the total diversity, especially since we did not search for parasites in extra gastrointestinal locations that may be shed in faeces. All sequencing primer sets have some level of bias and the small amount of sample from which DNA is extracted for PCR may simply not contain eggs or DNA of all parasites present (Elbrecht and Leese, 2015; Pawluczyk et al., 2015; Pompanon et al., 2012). This may explain why the 18S rRNA targeted high-throughput sequencing approach did not detect *Taenia* spp.; as well, taeniid egg shedding is sporadic, often shed in segments, and it is notoriously difficult to extract DNA from thick-walled taeniid eggs (Hidalgo et al., 2018). The same is true of *Baylisascaris* spp.; *Baylisascaris* eggs have thick walls, again making it difficult to liberate the DNA (Dangoudoubiyam et al., 2009; Testini et al., 2011). Taxonomic resolution from metabarcoding is limited by the availability of data in reference databases, which is often lacking for wildlife parasites. For example, unless the *Ascaris* sp. detected was a misidentification of *B. devosi* (which is possible considering that the 18S gene is highly conserved), the molecular methods used within this study failed to detect the species recovered by traditional methods. Additionally, because the 18S gene is highly conserved, it is important to interpret the metabarcoding results with caution. We report parasite taxonomy at genus level, as lower taxonomic classification is uncertain, but even so there are genera that may be misidentifications. For example, we detected a metastrongyloid lungworm that could be *Angiostrongylus gubernaculatus* or *Aelurostrongylus pridhami*, both previously identified in mustelids in North America (Anderson, 1963; Dougherty, 1946; Faulkner et al., 2001), or a closely related genus not represented in the reference databases we used. Further, it is important to note that the parasites detected here through 18S rRNA high-throughput sequencing may not be parasites of wolverines, but instead may be a parasite of an infected prey item (Sheppard et al., 2005; reviewed in Pompanon et al., 2012).

The molecular methods used here allow us to comprehensively characterise the broader parasite diversity in wolverines. However, due to the lower prevalence of parasites detected using molecular methods (22% total; no more than 13% prevalence for any given parasite) and because of the added benefit of abundance data associated with traditional methods, only the data obtained from traditional techniques was used when running our models. As reported previously (Addison and Boles, 1978), adult helminths *Baylisascaris devosi* and *Taenia* spp., including *T. twitchelli*) dominated the gastrointestinal tract helminth fauna of wolverines, present in 72% and 22% of wolverines in the current study, respectively, compared to 74% and 11%, respectively, in Addison and Boles (1978). Compared to previous studies, we found lower prevalence (2%, n = 1) of *Sarcocystis* (80%) and *Trichinella* (88%) (Dubey et al., 2010; Reichard et al., 2008b), although the last was based on larval recovery from muscle rather than intestinal based methods (Reichard et al., 2008b). It is likely that the DNA we detected for *Trichinella* is either from larvae ingested from a prey item, or from a transient adult nematode. The high parasite prevalence detected in our wolverine samples using count methods may reflect the fact that wolverines travel huge distances across a heterogeneous geographic range, from boreal forests to the Arctic tundra (Copeland et al., 2010; Dawson et al., 2010), which may lead to high exposure to parasites. It is more likely, however, that diet drives the high parasite prevalence detected. Wolverines consume an intensely varied diet of live prey and carcasses (Lofroth et al., 2007; Koskela et al., 2013), which may lead to elevated infection rates from trophically transferred parasites, such as *B. devosi* (transmitted directly and through paratenic hosts) and *T. twitchelli* (transmitted through consumption of intermediate hosts, including ground squirrels (family: Sciuridae), lemmings (family: Cricetidae), voles (family: Cricetidae), muskrats (*Ondatra zibethicus*), and porcupine (*Erethizon dorsatum*)) (Rausch, 1959).

In total, 10 species of *Baylisascaris* exist worldwide, most of which utilise carnivorous mammals as definitive host, with a smaller prey host serving as a paratenic host (Sapp et al., 2017). Some species of *Baylisascaris* incur detrimental health effects on their paratenic hosts; the raccoon roundworm, *B. procyonis*, for example, causes severe or even fatal neurological disease in humans and wildlife, yet little or no clinical disease in raccoon definitive hosts (Sapp et al., 2017). The effect that *B. devosi* infection has on wolverine health remains unknown; however, as they serve as definitive hosts, it is likely to be minimal. The common occurrence of *Baylisascaris* in species of the lower Arctic is attributed to the parasite's ability to persist in the external environment. It would appear, however, there is perhaps a northern limit of *B. devosi*, indicative from our finding that *B. devosi* abundance decreases with latitude –an important finding considering that increasing temperatures within the Arctic are expected to contribute to the northern expansion of parasites into regions that were previously inhospitable (Brooks and Hoberg, 2007; Kutz et al., 2009; McLaughlin, 2011). This finding resembles what is seen in some ascarid nematodes, such as *Toxocara canis* which does poorly above 60°N (reviewed by Jenkins et al., 2011), but not others, such as *Toxascaris leonina* which is found all the way up in to the high Arctic (Andreassen et al., 2017; Kapel and Nansen, 1996). Lindenfors et al. (2007) found latitude to be a primary predictor of parasite species richness in carnivores. *Taenia* spp. have a broad host range in mammalian definitive hosts that occupy northern territories, including brown bears (*Ursus arctos*), wolves (*Canis lupis*), reindeer (*Rangifer tarandus*) and arctic foxes (*Vulpes lagopus*) (Kapel and Nansen, 1996; Lavikainen et al., 2011). The prevalence of *Taenia* spp. in northern environments is again likely owed to the ability of *Taenia* eggs and gravid proglottids to survive for months in the external environment (Ilsøe et al., 1990), and the ability to transmit between predator-prey cycles. A likely intermediate host for *T. twitchelli* consumed by wolverine in Nunavut include ground squirrels, lemmings, voles, muskrats, and porcupine (*Erethizon dorsatum*; Rausch 1954; Dalerum et al., 2009; Kukka and Jung 2015).

Our findings showed that the abundance of *B. devosi*, total parasite abundance of both *Baylisascaris* and *Taenia*, and co-infection of *Baylisascaris* and *Taenia* (i.e. 0, 1 or ≥2 parasite species present) did not differ with sex or age class, a finding that mirrors what is seen with *Sarcocystis* and *T. gondii* infections in wolverines (Dubey et al., 2010; Reichard et al., 2008a). The lack of sex bias, however, challenges what might be expected, as male wolverines occupy a larger home range compared to females (Pasitschniak-Arts and Larivière, 1995) which may increase their exposure to parasites. Geographical range size is also considered an important determinant of parasite infection in various other carnivores (Lindenfors et al., 2007). Additionally, male wolverines are larger in both size and mass compared to females (Awan and Szor, 2012; Banci, 1994); larger-bodied organisms require a greater resource intake, potentially increasing exposure to trophically transmitted parasites (reviewed in Morand and Poulin 1998). Alternatively, the increased parasite abundance typically found in males compared to females may be attributable to immunological differences that exist between sexes, which may in turn influence the susceptibility of male hosts (reviewed in Klein 2004).

Wolverines are a culturally important species to northern communities and, as such, it is important to address parasite species that are of concern to human health. It is unlikely that the parasites detected within this paper are of risk to trappers and hunters handling wolverine carcasses. *Trichinella* spp. are zoonotic, but it is important to note that the *Trichinella* spp. detected within our study may be from an ingested prey item, rather than being a true parasite of wolverines, and so may not pose a risk.

Monitoring wildlife plays an important role in identifying changes such that actions can be taken to mitigate or minimize pressure. Here we have filled a knowledge gap in parasite community data and have shown, through the use of combined adult parasite recovery and molecular methods, that the parasite diversity of wolverines is greater than previously observed. To this end, we recognise the value of molecular methods to aid adult parasite recovery, especially in remote species. We encourage more work be done to improve the advancement of molecular screening methods for parasites, including broader databases with sequences from morphologically confirmed specimens, and interpretation of findings in light of the best available understanding of parasite life cycles and known host and geographic distributions.

## Ethical statement

The authors declare that they have no conflicts of interest. Wolverines are traditionally harvested by Inuit in Nunavut; a subsample of the harvest was collected with the return of carcasses to the Government of Nunavut offices.

## Declaration of competing interest

None.
