# Supplementary material for: Parasites of an Arctic scavenger; the wolverine (Gulogulo)
Source: Int J Parasitol Parasites Wildl. 2020 Oct 16;13:178–85. doi: 10.1016/j.ijppaw.2020.10.004 (PMC7591336; doi:10.1016/j.ijppaw.2020.10.004)
Supplement: Multimedia component 1 [file mmc1.docx]

**Supplementary Table 1.** Infection prevalence for all six parasites detected in 54 wolverines when using 18S high-throughput sequencing. Due to discrepancies in taxonomic identification between SILVA and NCBI databases, parasites identifications are stated at the closest taxonomic rank that encompasses the identifications given in both databases. Prevalence is show against associated host and geographic metadata (where AR = Arviat, BL = Baker Lake, CB = Cambridge Bay, KU = Kugluktuk, and RB = Repulse Bay (Naujaat); see Figure 1 for locations). For age class, A = Adult, J = Juveniles, Y = yearling.

| **Parasite genera** | **ID** | **Location** | | | **Sex** | **Age class** | | **Age (Years)** | | **Length (cm)** | **Mass (Kg)** |
| --- | --- | --- | --- | --- | --- | --- | --- | --- | --- | --- | --- |
| Order: Ascaridida | 314 | BL | | | M | Y | | | 1 | 858 | 10.9 |
|  | 1793 | BL | | | F | J | | | 0 | 742 | 8.2 |
|  | 2924 | CB | | | F | J | | | 0 | 728 | 9.9 |
|  | 3305 | AR | | | F | Y | | | 1 | 390 | 10.0 |
|  | 4031 | CB | | | M | Y | | | 1 | 788 | 13.1 |
|  | 664 | BL | | | F | J | | | 0 | 690 | 8.2 |
|  | 716 | AR | | | F | A | | | 8 | 720 | 7.2 |
|  |  |  | | |  |  | | |  |  |  |
| Family: Angiostrongylidae | 2507 | KU | | | F | J | | | 0 | 789 | 8.5 |
|  |  |  | | |  |  | | |  |  |  |
| *Crenosoma* sp. | 3740 | KU | | | F | A | | | 7 | 757 | 10.3 |
|  |  |  | | |  |  | | |  |  |  |
| Subclass: Eucestoda | 35 | AR | | | M | A | | | 2 | 814 | 12.3 |
|  |  |  | | |  |  | | |  |  |  |
| *Sarcocystis* sp. | 612 | BL | | | M | A | | | 2 | 875 | 13.7 |
|  |  |  | | |  |  | | |  |  |  |
| *Trichinella* sp. | 23 | AR | | | M | A | | | 2 | 804 | 12.1 |
|  |  |  |  |  | |  |  | | | |  |
